# Ionic release and electrochemical testing of 3-D printed orthodontics metallic bands made of Co-Cr and stainless-steel alloys

**DOI:** 10.1093/ejo/cjaf050

**Published:** 2025-07-05

**Authors:** Spiros Zinelis, Aikaterini Sakellari, Sotirios Karavoltsos, Georgios Polychronis, Nearchos Panayi, Spyridon N Papageorgiou, Theodore Eliades

**Affiliations:** Department of Biomaterials, School of Dentistry, National and Kapodistrian University of Athens, Athens, Greece; Laboratory of Environmental Chemistry, Department of Chemistry, National and Kapodistrian University of Athens, Athens, Greece; Laboratory of Environmental Chemistry, Department of Chemistry, National and Kapodistrian University of Athens, Athens, Greece; Department of Biomaterials, School of Dentistry, National and Kapodistrian University of Athens, Athens, Greece; Department of Dentistry, European University Cyprus, Nicosia, Cyprus; Clinic of Orthodontics and Pediatric Dentistry, Center for Dental Medicine, University of Zurich, Switzerland; Clinic of Orthodontics and Pediatric Dentistry, Center for Dental Medicine, University of Zurich, Switzerland

**Keywords:** corrosion, Co-Cr alloys, stainless steel alloys, 3D printing, orthodontic bands

## Abstract

**Objective:**

The aim of this study was to investigate the electrochemical characteristics and ionic release of three-dimensional (3D) printed orthodontic metallic bands made of stainless-steel (SS) and Cobalt-Chromium (Co-Cr) alloys.

**Materials and Methods:**

Thirty-six orthodontic metallic bands were prepared by 3D printing, employing SS (n = 18) and Co-Cr (n = 18) alloys. The ionic release from each alloy was determined according to ISO 27020:2019 requirements employing flame atomic absorption spectrometry (FAAS). The electrochemical properties were characterized by Open Circuit Potential (OCP), Anodic Scan (AS) and Electrochemical Impedance Spectroscopy (EIS). In all cases a 0.1M NaCl, 0.1 M lactic acid solution was used. The EIS data were fitted to a Randles circuit and the sum of ionic release of probed elements after immersion along with OCP, zero corrosion potential (E_corr_), corrosion current density (I_corr_), and pitting corrosion potential (E_pit_) were compared with t-tests/Mann-Whitney tests (α = 0.05).

**Results:**

SS bands showed an approximately 44-fold higher total ionic release compared to Co-Cr ones. Electrochemical testing revealed no statistically significant differences for E_corr_ but Co-Cr illustrated more beneficial OCP, I_corr_ and E_pit_ values than SS. Both alloys illustrated semicircles in Nyquist plots, implying that the electrochemical process was under charge transfer control, while the equivalent circuit simulation showed a higher charge transfer resistance for Co-Cr alloy (SS:14191.1 *vs* Co-Cr:33158.7 Ωcm^2^), implying lower corrosion current and thus decreased corrosion rate.

**Conclusion:**

Immersion and electrochemical testing indicated that Co-Cr alloys showed increased corrosion resistance over SS ones, which might affect efficacy under clinical use.

## Introduction

In orthodontics, the use of metallic bands for molars is considered by many clinicians as an integral part of fixed-appliance orthodontic treatment [1]. These bonded or cemented bands provide a stable tooth anchorage enabling a variety of orthodontic movements [2, 3], might reduce demineralization, and are less prone to bonding failure compared to bonded brackets / tubes [4–6]. However, their placement requires prolonged chair time and might be associated with patient discomfort during adaptation to the tooth’s shape [6, 7]. In addition, the need to have a large number of band sizes in order to select the proper one might have financial inventory implications. To address these issues, three-dimensional (3D) printed technology has emerged as a highly promising fabrication technique for in-office metallic band production, with 3D printers utilizing a high energy source capable of melting metal powder. Such bands are constructed layer by layer, based on digital 3D tooth models [8–10]. These custom-made bands thus fit precisely to the patient’s teeth and tend be associated with less discomfort and more cost-effective provided that they are manufactured in dental labs Unlike the commercially available bands that are mainly made of stainless steel (SS) alloys [11], 3D printed bands may comprise a variety of alloys apart from SS, including Cobalt Chromium (Co-Cr) alloys.

Introduction of such innovative devices to clinical use requires a thorough investigation of a spectrum of different properties (i.e. biocompatibility, corrosion resistance, mechanical properties) to ascertain patient safety. Corrosion resistance especially is of paramount importance since the intraoral area is a highly demanding environment in terms of corrosion, related to multiple fluctuating variables including pH, temperature, salivary flow, masticatory forces, food / fluid intake, microbial flora, tooth brushing, and mouthwash use [12–19]. Such 3D printed bands are hence subject to frequent corrosion incidents, with their resistance depending on factors such as alloy consistency, phase distribution, grain size, porosity, surface geometry, and roughness [20–23]. Protective passivation layers consisting of metal oxides are often not resistant to the aggressiveness of the intraoral conditions. Therefore, subsequent metal ions’ release may exert adverse toxic effects and allergic reactions highlighting biocompatibility issues [23–25], as in the case of SS alloys, which are prone to corrosion under acidic conditions and in chloride-containing media [26, 27], releasing nickel (Ni), iron (Fe), or chromium (Cr) ions to adjacent tissues and saliva [28], with possible biological consequences. In the case of Co-Cr alloys, Cobalt (Co) has been associated with genotoxic and carcinogenic activity [29, 30], while in case of SS alloys, Ni and Cr have been associated with allergic and cytotoxic reactions, respectively [31–33]. On the other hand, metallic mass loss due to ionic release may undermine structural durability or performance and longevity of any metallic structure [18, 34, 35], with 3D printed bands not constituting an exception to this rule.

A considerable amount of data is available in the literature on the electrochemical behaviour and ion release of commercially available SS orthodontic bands [25, 33, 36, 37], with their majority being cast-fabricated. Highly affected by the manufacturing method [22], the surface phenomenon of corrosion is aggravated by increased roughness and porosity, which generally deteriorate corrosion resistance [22, 23]. The 3D printing of molar bands may alter their electrochemical behavioural properties, albeit this has not been so far tested.

Therefore, the aim of this study was to investigate the electrochemical characteristics and ionic release of 3D printed orthodontic metallic bands made of SS and Co-Cr alloys. The null hypothesis is that no significant differences between SS and Co-Cr alloys exist in their electrochemical properties.

## Materials and methods

### Specimen preparation

Thirty-six orthodontic metallic bands, employing SS (n = 18) and Co-Cr (n = 18) alloys, were designed by the 3D Computer-Aided Design (CAD) software Meshmixer (Autodesk, San Rafael, Calif, USA) and fabricated by 3D printing. The Phenix system PSX 100 (3D systems, Rock Hill, South Carolina, USA) was used to print the metallic bands using Biosint Co-Cr powder alloy (Stroumbos, Athens, Greece) with 16 μm mean particle size and nominal composition %w/w: Co 63.0, Cr 24.0, W 8.0, Mo 3.0, Si 1.0 and Nb 1.0. For the SS alloy the EP-M150 Metal Dental 3D Printer (EPlus3D, Zhejiang, China) was used, with a 316L/ 1.4404 SS alloy (Eplus3D SS 316L). The nominal composition of 316L / 1.4404 is %w/w: Fe balance, Cr 16.5-18.5, Ni 10.0—13.0, Mo 2.0-2.5, Mn 2.0, Si 1.0, P, S, N and C < 0.5. All devices were heat-treated for 2 hours at 900°C.

### Solution preparation

The procedures of sampling, immersion, and testing were carried out according to ISO 27020:2019 [38] and ISO 10271:2011 [39]. An aqueous solution comprising 0.1 mol/L lactic acid and 0.1 mol/L NaCl was prepared immediately before use. Lactic acid (C3H6O3) 90% (10.0 ± 0.1 g) and NaCl (5.85 ± 0.005 g) were initially dissolved in approximately 300 mL of ultrapure water of 18.2 MΩ cm (Millipore, Bedford, MA, USA), transferred to a volumetric flask (ISO 10271 par 4.1.4.6) and filled up to 1L. The pH was checked and complied with ISO requirements (2.3 ± 0.1).

### Static immersion

The experimental part was carried out according to ISO 10271:2011 (par 4.1.6.1.2). A set of 2 metallic bands (1 clinical case) was placed in six containers with their bonding surfaces facing down in contact with the container. In all cases, 50 mL of solution were added, and each container was checked to ensure that there is no contact between the metallic devices, while an empty container filled with the same solution volume served as reference sample. All containers were sealed and stored at 37 ± 1°C for 7 days. Quantification of Fe, Ni, Cr and Co in the solutions was performed with Atomic Absorption Spectrometry (AAS), following appropriate dilution with ultrapure water.

All glassware used was previously washed thoroughly, soaked in dilute HNO_3_ (Merck, Darmstadt, Germany), and rinsed with ultrapure water of 18.2 MΩ cm (Millipore). For the preparation of all required solutions, class A volumetric glassware was used. Calibration standard solutions were prepared by Fe, Co, Cr and Ni stock standard solutions (Merck, Darmstadt, Germany), appropriately diluted with ultrapure water.

Analysis of Fe was carried out by flame atomic absorption spectrometry (FAAS) in an AAS unit (SpectrAA 200; Varian, Mulgrave, Victoria, Australia) under the following conditions: wavelength 248.3 nm, slit width 0.2 nm, acetylene-air oxidizing flame, 5 mA lamp current, and limit of detection 0.2 mg L^-1^. For Co, Cr and Ni, the analyses were performed by graphite furnace atomic absorption spectrometry (GFAAS) with Zeeman background correction, employing a SpectrAA 640Z unit (Varian, Mulgrave, Victoria Australia), under the following conditions: for Co: wavelength 242.5 nm, spectral bandwidth 0.2 nm, 7 mA lamp current, pyrolysis temperature 750 ^o^C, atomization temperature 2300 ^o^C, limit of detection 0.8 μg L^-1^; for Cr: wavelength 357.9 nm, spectral bandwidth 0.2 nm, 7 mA lamp current, pyrolysis temperature 1000 ^o^C, atomization temperature 2600 ^o^C, limit of detection 0.5 μg L^-1^; for Ni: wavelength 232.0 nm, spectral bandwidth 0.2 nm, 4 mA lamp current, pyrolysis temperature 800 ^o^C, atomization temperature 2400 ^o^C, limit of detection 0.8 μg L^-1^.

### Electrochemical testing: Open Circuit Potential (OCP), Anodic Scan (AS), and Electrochemical Impedance Spectroscopy (EIS)

**Figure 1.**
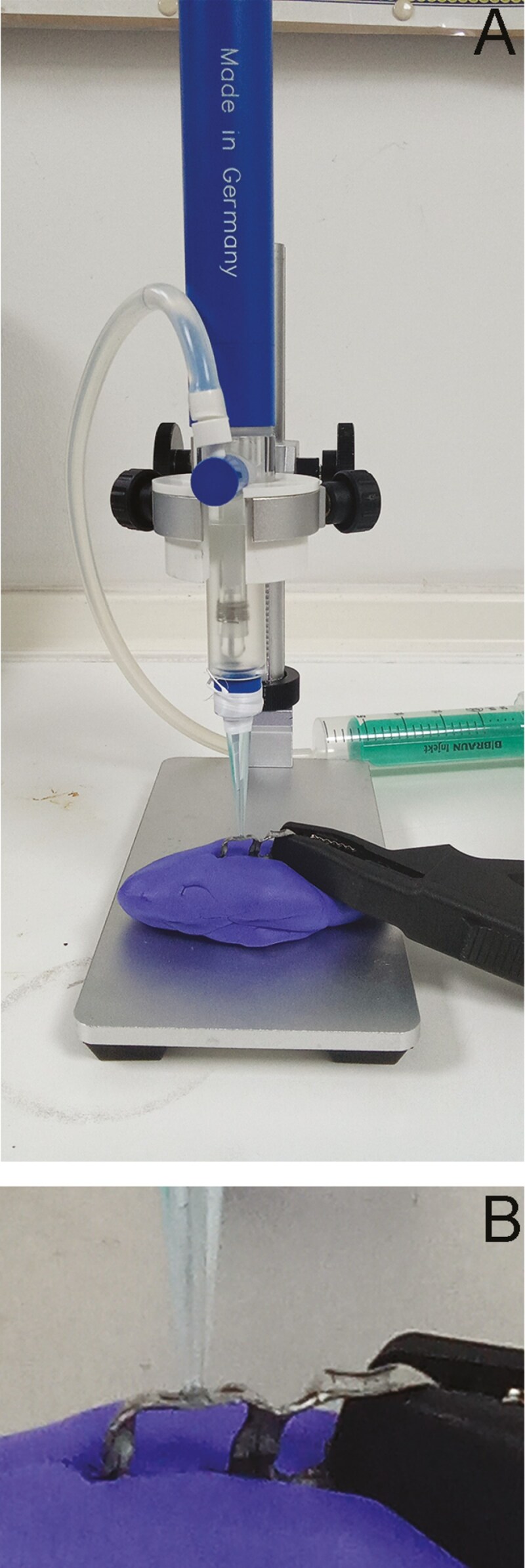
A) Mini Cell System consists of a Saturated Calomel Electrode (SCE) and a Pt wire counter electrode while the tested pieces are the working electrode. B) A close view of the probe transferring the solution onto the tested surface of the working electrode (orthodontic band).

The electrochemical properties of each group were identified through direct measurements on band surfaces, employing an electro chemical Mini Measuring Cell MMC (Ibendorf & Co. Berlin, Germany). Six metallic bands from each group were used as the working electrode of the system, while a Saturated Calomel Electrode and a Platinum (Pt) electrode were used as reference and measuring electrodes, respectively (Fig. 1). The MMC was filled with electrolyte (0.1M NaCl and 0.1M Lactic acid) and the electrode was connected with a potensiostat / galvanostat EmStat4s (PalmSens, Houten, The Netherlands), controlled by the dedicated software PsTrace vs 5.10 (PalmSens, Houten, The Netherlands). OCP was recorded versus time for 30 min and then EIS spectra were acquired in a frequency range from 10^5^ Hz to 10^-2^ Hz, with 10 points per decade and 10 mV wave amplitude. All measurements were conducted at OCP and 100 μA current range. The real (Z’) and imaginary parts (Z’’) of impedance were recorded at each frequency and thus absolute impedance (Z) and phase angle were determined to construct Nyquist and Bode plots. Equivalent circuit fitting was performed employing the same software (PsTrace). The equivalent circuit (Randles cell) consists of two resistors in series (solution resistance [Rs] and charge transfer / corrosion resistance [Rc]) and a Constant Phase Element module (CPE), which models the behaviour of the double layer. The CPE component is considered as an imperfect capacitor. Following EIS testing, anodic scan curves were recorded starting from -0.5V up to 1.5V with 1 mV/s sweep rate. Butler-Volmer fit was used to identify zero current potential (E_corr_) and corrosion current density (I_corr_), while pitting corrosion potential (E_pit_) was also recorded from the curve. The contact area of the tip was calculated employing a steromicroscope (0.0055cm^2^) and was used for the calculation of current density.

### Statistical analysis

After normality testing with the Shapiro-Wilk test, descriptive statistics were calculated, consisting of means with Standard Deviations (SD) (or medians with Interquartile Ranges [IQR] for skewed data). Differences between groups were calculated with independent samples t-tests (or Mann-Whitney tests for skewed data). Chi-squared tests were used to assess fitting in the Randles circuit. All analyses were performed in StataSE 18.5 (StataCorp, College Station, TX, USA) with a two-sided α of 5% and an open dataset available through Zenodo (https://doi.org/10.5281/zenodo.15173283).

## Results

### Immersion testing

Table 1 indicates the results of the quantitative analysis of Fe, Co, Cr and Ni, along with their total values according to ISO 27020:2019 [38] and 10271:2011 (par 4.1) [39]. SS bands showed an approximately 44-fold increase in total ionic release compared to Co-Cr ones (P < 0.001), together with higher variability (as seen by the increased SDs and coefficients of variation [CV]).

**Table 1.** Descriptive statistics for the ionic release of Fe, Cr, Ni and Co and sum values calculated for both materials tested. The unit of mean and sum values represents the ionic release for 1case in μg/7 days/L (ppb). (n = 6 cases, 2 bands for each case).

Elements	SS		Co-Cr		
	Mean (SD)	CV	Mean (SD)	CV	P value^*^
Fe	637774 (213727)	0.34			-
Cr	41826 (12328)	0.29	1532 (109)	0.07	<0.001
Ni	209531 (62716)	0.30			-
Co			18156 (2048)	0.11	-
Sum	889130 (287185)	0.32	19688 (2145)	0.11	<0.001

^*^from independent samples t-test.

Co-Cr, Cobalt-Chromium; CV, coefficient of variation; SD, standard deviation; SS, stainless steel.

### Electrochemical testing: OCP, AS, and EIS

**Figure 2.**
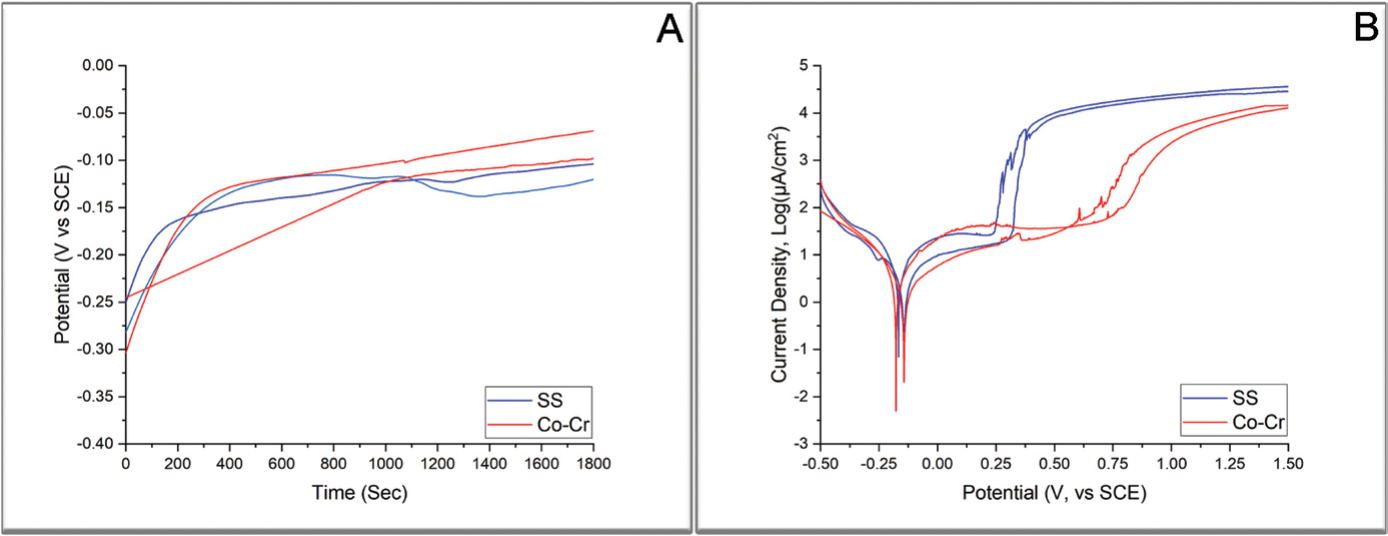
Open Circuit Potential Curves (A) and anodic scan curves (B) from both alloys tested.

Fig. 2A presents obtained OCP curves, while the measured final OCPs are reported in Table 2. All curves shifted towards a higher potential with a high rate over the first 15 min and a low one for the rest of the monitoring time. Fig. 2B shows the anodic scan polarization curves, while the numerical results from both methods are presented in Table 2. Statistically significant differences were identified for OCP, I_corr_, and E_pit_ between the materials tested.

**Table 2.** Descriptive statistics for OCP, E_corr_, I_corr_ and E_pit_ and between-group differences (n = 6). All potentials are versus SCE.

Group	OCP (V)	E_corr_ (V)	I_corr_ (μA/cm^2^)	E_pit_ (V)
Statistic	Median (IQR)	Mean (SD)	Mean (SD)	Mean (SD)
SS	−0.09 (-0.10, -0.07)	−0.19 (0.04)	35.50 (9.66)	0.25 (0.15)
Co-Cr	−0.10 (-0.12, -0.10)	−0.20 (0.05)	20.77 (8.55)	0.49 (0.11)
Test	Mann-Whitney	Ind. t-test	Ind. t-test	Ind. t-test
P value	0.03	0.56	0.01	0.01

Co-Cr, Cobalt-Chromium; Ind., independent-samples; IQR, interquartile range; SD, standard deviation; SS, stainless steel.

**Figure 3.**
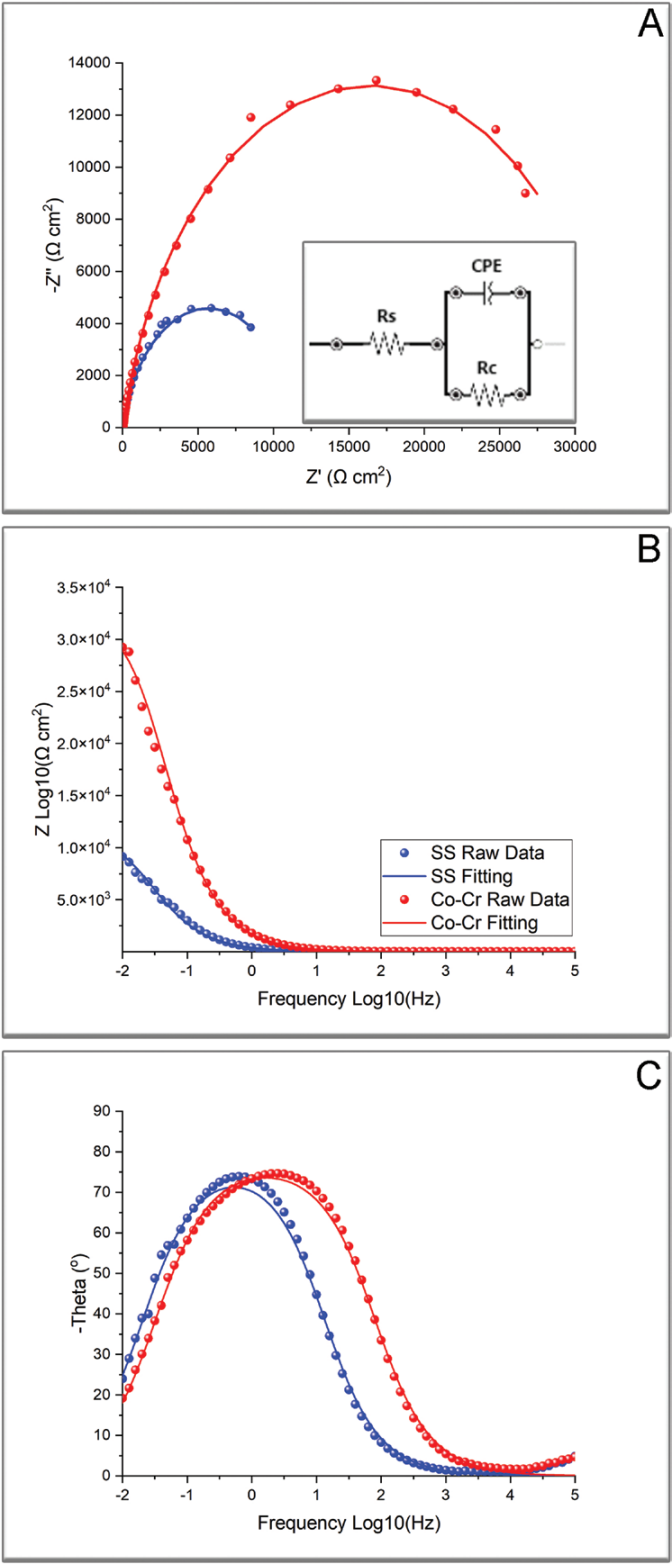
A) Nyquist curve with Real (Z’) and Imaginary (Z’’) components of impedance. Inset: The Randlers cell with omic (Rs and Rc) and capacitor resistance (CPE) elements B) Bode plot of impedance magnitude over frequence and C) Bode plots of phase angle. Legend for dots and lines in B.

Nyquist plots and Bode plots are presented in Fig 3. Fig 3A shows the Nyquist plot where Co-Cr demonstrates a larger semicircle compared to that of SS. Fig. 3B and 3C demonstrate Bode plots for impedance and phase angle respectively, with Co-Cr showing increased impedance compared to that of SS in low frequencies (Fig 3B). The inset in Fig 3A demonstrates the Randles equivalent circuit. All characteristics for circuit elements are reported in Table 3, along with Chi-squared values, indicative of fitting.

**Table 3.** Impedance parameters obtained by data fitting with the Randles electrical equivalent circuit along with Chi-Squared values.

	SS	Co-Cr
Rs (Ω cm^2^)	42.3	39.4
Rc (Ω cm^2^)	14191.1	33158.7
Q (μF cm^-2^s^n^)	300.4	120.5
n	0.88	0.85
Chi-Squared	0.0026	0.0032

Co-Cr, Cobalt-Chromium; Rc, charge transfer; Rs, solution resistance; SS, stainless steel.

## Discussion

The experimental data of this study rejected the null hypothesis for many of the studied variable, since significant differences were identified employing electrochemical testing between SS and Co-Cr orthodontic bands.

The tested SS bands demonstrated an ionic release exceeding that of Co-Cr by an order of magnitude, raising concerns about the dilution of metals (Fe, Cr and Ni) under intraoral conditions. However, no other requirements have been set by ISO [38], other than the confirmation of the results with the arbitrary value provided by the manufacturer. Therefore, comments about the safety of these devices are not feasible. Contrary to all other ISO standards related with corrosion resistance where the results are provided as μg per surface, in the case of bands tubes and brackets the results are given as mg per case hindering the comparison with data from literature. The amount of leached elements (Table 1) did not match their concentrations in the SS alloy, since Ni with almost half of Cr content showed an almost four-fold ionic release, implying a non-uniform corrosion mechanism. Although the SD is used to characterize the dispersion of a population relatively to its mean value, its use in the comparison of the variability among different data sets, with great differences in their means is not feasible, as herewith, calculating for this purpose the CV. Data in Table 1 clearly depict that all probed elements of SS group demonstrated considerably high CVs, implying that the tested bands showed significant differences among each other, in terms of ionic release. This could be attributed to inhomogeneous microstructure, partial fusing of metal powder, inappropriate selection of operating parameters, or other complications of 3D printing technology [40].

Contrary to traditional electrochemical testing, where the specimens are embedded in acrylic resin and submerged in tested solutions, the employed Mini Cell System (MCS) provides the capacity of direct measurement in the final products, accurately simulating exposure to oral fluids. The specific setup characterizes the medical device as a final product (including surface characteristics and other properties), instead of the material of which it is constructed.

The OCP increased towards anodic values, implying the formation of a passive film in the tested solution for both alloys. The OCP found for Co-Cr alloys in the present study is comparable to values previously reported in the literature, approximating -0.17V [41, 42]. The finding that both alloys share relatively similar OCP values may lessen concern for galvanic action [23], in case bands of different alloys are placed to the same patient. Following matching of OCP, the tested alloys demonstrated similar E_corr_ (Table 2), a value denoting the ionization tendency of alloys tested against a specific reagent. This tendency is decreased towards higher E_corr_ values. The E_corr_ values found in the present study are likewise similar to those previously reported in the literature (-0.19V) [43] for SS and within the range (-0.25V, -0.16V) for dental porcelain fused to metal Co-Cr alloys [44]. On the other hand, Co-Cr alloys showed significantly lower I_corr_ (Table 2) values, implying a lower corrosion rate compared to SS ones. E_pit_ indicates the lowest potential at which pitting or crevice corrosion initiates, implying collapse of the oxide film protective action. The values found in the present study are similar to those previously reported for SS alloys (0.28V) [43], albeit exceeding the range reported for Co-Cr for dental cast alloys (0.61V~ 0.70V) [44], potentially appended to differences in the elemental composition and/or microstructure between cast and 3D printed alloys [45]. Based on E_pit_ values, the oxide film of SS alloy demonstrated inferior protection in pitting and crevice corrosion and should be thus considered as more vulnerable to corrosion phenomena under the acidic conditions tested.

Both Nyquist curves (Fig. 3A) showed semicircles, implying that the electrochemical process was under charge transfer control. The equivalent circuit simulation showed a lower charge transfer resistance for SS alloy (Rc: 14191.1 vs 33158.7 Ωcm^2^), implying higher corrosion current and thus increased corrosion rate. In addition, Bode plots (Fig. 3B) showed at low frequencies a higher impedance modulus for Co-Cr alloy, indicating a higher corrosion resistance compared to that of SS. The solution resistance (Rs) was estimated close to previously reported values (32.5 ~35.9 Ωcm^2^), while both Chi-Squared values are close to zero, indicating a satisfactory fitting of experimental data (Table 3).

This study is not free from the inherent limitations of electrochemical and immersion testing, since intraoral corrosion occurs through the developed biofilm. However, it is widely accepted that experimental setups cannot fully simulate oral conditions. According to ISO, ionic release is performed in a very acidic solution with pH 2.3, far from the normal pH of the human cavity, thus direct extrapolation of the results to clinical practice, as a routine estimation of ionic release, is not feasible. Nonetheless, the results of this study confirm that Co-Cr alloys has a much better corrosion resistance compared to SS alloys, although the aetiology behind this finding should be further tested.

## Conclusion

Under the limitations of this study, significant differences in the electrochemical and ionic release properties of Co-Cr and SS alloys used for 3D printing of orthodontic bands. However, whether this can impact their biocompatibility or their clinical use intraorally remains unclear.

## Data Availability

The data in the current study are openly available (https://doi.org/10.5281/zenodo.15173283).
